# Cardiovascular rehabilitation soon after stroke using feedback-controlled robotics-assisted treadmill exercise: study protocol of a randomised controlled pilot trial

**DOI:** 10.1186/1745-6215-14-304

**Published:** 2013-09-22

**Authors:** Oliver Stoller, Eling D de Bruin, Corina Schuster-Amft, Matthias Schindelholz, Rob A de Bie, Kenneth J Hunt

**Affiliations:** 1 Department of Engineering and Information Technology, Institute for Rehabilitation and Performance Technology, Bern University of Applied Sciences, Pestalozzistrasse 20, 3400, Burgdorf, Switzerland; 2 Department of Epidemiology, Maastricht University and Caphri Research School, P.O. Box 616, 6200, MD, Maastricht, The Netherlands; 3 Reha Rheinfelden, Salinenstrasse 98, 4310, Rheinfelden, Switzerland; 4 Department of Health Sciences and Technology, Institute of Human Movement Sciences and Sport, ETH Zurich, Wolfgang-Pauli-Strasse 27, 8093, Zurich, Switzerland

**Keywords:** Stroke, Aerobic exercise, Sub-acute, Non-ambulatory, Aerobic capacity, Robotics-assisted

## Abstract

**Background:**

After experiencing a stroke, most individuals also suffer from cardiac disease, are immobile and thus have low endurance for exercise. Aerobic capacity is seriously reduced in these individuals and does not reach reasonable levels after conventional rehabilitation programmes. Cardiovascular exercise is beneficial for improvement of aerobic capacity in mild to moderate stroke. However, less is known about its impact on aerobic capacity, motor recovery, and quality-of-life in severely impaired individuals. The aim of this pilot study is to explore the clinical efficacy and feasibility of cardiovascular exercise with regard to aerobic capacity, motor recovery, and quality-of-life using feedback-controlled robotics-assisted treadmill exercise in non-ambulatory individuals soon after experiencing a stroke.

**Methods/Design:**

This will be a single-centred single blind, randomised control trial with a pre-post intervention design. Subjects will be recruited early after their first stroke (≤20 weeks) at a neurological rehabilitation clinic and will be randomly allocated to an inpatient cardiovascular exercise programme that uses feedback-controlled robotics-assisted treadmill exercise (experimental) or to conventional robotics-assisted treadmill exercise (control). Intervention duration depends on the duration of each subject’s inpatient rehabilitation period. Aerobic capacity, as the primary outcome measure, will be assessed using feedback-controlled robotics-assisted treadmill-based cardiopulmonary exercise testing. Secondary outcome measures will include gait speed, walking endurance, standing function, and quality-of-life. Outcome assessment will be conducted at baseline, after each 4-week intervention period, and before clinical discharge. Ethical approval has been obtained.

**Discussion:**

Whether cardiovascular exercise in non-ambulatory individuals early after stroke has an impact on aerobic capacity, motor recovery, and quality-of-life is not yet known. Feedback-controlled robotics-assisted treadmill exercise is a relatively recent intervention method and might be used to train and evaluate aerobic capacity in this population. The present pilot trial is expected to provide new insights into the implementation of early cardiovascular exercise for individuals with severe motor impairment. The findings of this study may guide future research to explore the effects of early cardiovascular activation after severe neurological events.

**Trial registration:**

This trial is registered with the Clinical Trials.gov Registry (NCT01679600).

## Background

Cardiovascular-related diseases are the leading causes of death [1] and long-term disability worldwide [2]. Stroke affects about 15 million people each year, whereby 5 million die and another 5 million remain permanently disabled [3]. It has been estimated that stroke will occur in 35% of the population over the age of 65, a group that will increase in proportion due to demographic shifts in most populations [4]. This leads to an increased need for effective rehabilitation programmes to enhance recovery, improve functional status and quality-of-life, while considering the future challenges in worldwide health care economics [5].

To date, stroke rehabilitation research has focussed primarily on restoring motor control to promote independent function during daily life. However, around 75% of post-stroke individuals suffer from cardiac disease [6,7], and the majority have low endurance for exercise as a consequence of immobility [8]. Previous work has shown that peak oxygen uptake (VO_2_peak) is reduced to 10 to 17 mL/min/kg within the first 30 days after stroke [9-11] and does not rise over 20 mL/min/kg after 6 months [11-14]. These values are 25 to 45% lower than VO_2_peak in age-matched healthy subjects [15,16]. This early and persistent decline in aerobic capacity can delay or inhibit participation in a therapeutic exercise programme, cause difficulties in the rehabilitation process and long-term post-stroke course of care, and limits an individual’s independent performance of functional activities [7,17].

In a recent meta-analysis it has been shown that cardiovascular exercise is beneficial for improving VO_2_peak and walking endurance in mild to moderately impaired individuals in sub-acute stroke (1 week to 6 months after onset) [18]. However, less is known about its impact on motor recovery, quality-of-life, and mortality. Previous trials did not consider post-stroke individuals with severe motor impairments due to the challenge of selecting appropriate training modalities and evaluation of effectiveness (for example, aerobic capacity). About 50% of stroke survivors may be non-ambulatory or even unable to walk a speed or distance necessary to achieve aerobic benefits [19]. This leads to inadequate training programmes and restricts the performance of valid cardiopulmonary exercise testing protocols. Given the fact that most motor recovery occurs in the first 3 months after a stroke [20], sub-acute rehabilitation programmes have to target neuromuscular recovery as well as cardiovascular exercise to optimize intervention outcomes. This could be achieved by the implementation of assistive devices to promote functional activities such as walking or stair climbing.

The application of robotics-assisted walking thus has substantial clinical potential. The technology offers several advantages over conventional rehabilitation strategies, such as motor function related training programmes within controlled evaluation settings, even for individuals with severe motor impairments. Robotics-assisted treadmill exercise (RATE), consisting of driven gait orthoses with an integrated body weight unloading system, opens new perspectives for cardiovascular exercise and evaluation of aerobic capacity in the early stages after stroke.

A novel assessment protocol has been developed to estimate key cardiopulmonary performance parameters during RATE [21,22]. This incorporates a human-in-the-loop feedback mechanism, which allows individuals to maximise their voluntary input and associated cardiovascular stress. A previous study provided evidence for the concept of feedback-controlled RATE (FC-RATE) to evaluate aerobic capacity and guide cardiovascular exercise in non-ambulatory individuals with sub-acute stroke [23]. Cardiopulmonary performance parameters yielded reasonable values following standardised exercise testing protocols. The next logical step is to evaluate the clinical efficacy and feasibility of the concept during early stroke rehabilitation. It has been hypothesised that FC-RATE is feasible and effective for maintenance or improvement of aerobic capacity in non-ambulatory individuals with sub-acute stroke. This might be highly relevant for further research to explore the association between cardiovascular rehabilitation and neural plasticity during early stroke rehabilitation.

This randomised controlled pilot trial aims: (1) to explore the clinical efficacy of early cardiovascular exercise on aerobic capacity, motor recovery, and quality-of-life; (2) to evaluate the feasibility of FC-RATE for cardiovascular rehabilitation in non-ambulatory individuals after stroke; and (3) to put forward recommendations for development and design of larger clinical trials regarding cardiovascular rehabilitation early after stroke.

## Methods/Design

All procedures involved in this trial will be conducted in compliance with national ethical standards and the Helsinki Declaration. Ethical approval has been obtained from the ethics committee of the canton of Aargau in Switzerland (Reference No. 2012/051).

### Design and setting

**Figure 1:**
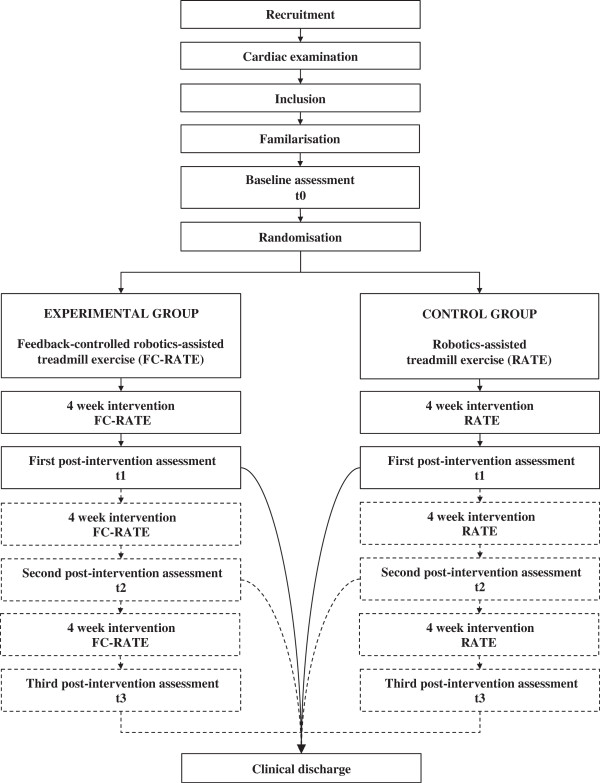
**Flow chart outlining the study protocol.** A 4-week intervention period includes baseline assessments (t0) and first post-intervention (t1) assessments (solid line). The dashed line represents a potential continuation of the intervention due to extended inpatient rehabilitation. Post-intervention assessments will be conducted after every 4-week intervention period and before clinical discharge.

This study is a single-centred single blind, randomised control trial with a pre-post intervention design. Subjects will be recruited at a neurological rehabilitation clinic in the northwestern part of Switzerland (Reha Rheinfelden). Figure 1 illustrates the plan of the study protocol.

### Eligibility criteria

Subjects will be enrolled after their first stroke if they are non-ambulatory. Inclusion criteria are: (1) clinical diagnosis of an initial stroke, (2) ≤20 weeks post-stroke at intervention onset, (3) age ≥18 years, (4) Functional Ambulation Category (FAC) ≤3, and (5) ability to understand the procedures and provide informed consent. Exclusion criteria are: (1) contraindications for cardiopulmonary exercise testing as outlined by the American College of Sports Medicine (ACSM) [24], (2) contraindications for RATE according to the device manufacturer, (3) concurrent neurological disease, (4) concurrent pulmonary disease, and (4) history of dementia.

### Recruitment

Potential subjects will be screened daily by searching the admission lists of the rehabilitation centre. The project leader will approach eligible subjects and explain the purpose of the study and ask whether potentially qualifying individuals have an interest in participating. If a potential subject expresses interest, the study will be explained in detail, all questions will be addressed and the subject will be invited to participate by providing signed informed consent. Subjects then will be referred for specific cardiac examination by a cardiologist to detect potential contraindications for cardiopulmonary exercise testing. The project leader and the responsible physician will make a final decision on inclusion.

### Randomisation, allocation concealment, and blinding

Subjects will be randomly allocated to an experimental group (FC-RATE) or to a control group (RATE). An independent researcher will generate a 4-block randomisation list and send it to the clinic's pharmacy for safekeeping. The leader of the physical therapy team will call the pharmacy to request a subject’s allocation. Only the treating therapists will have access to the allocated documents. The project leader, who is blind for allocation of the subjects, will carry out all testing procedures as an independent examiner. A further research assistant will be responsible for data input. Subjects will be told not to talk to the examiner about the group allocation or therapy content during the post-intervention assessments. Randomisation will be concealed to the independent examiner until the last post-intervention assessment has been performed. The data will be analysed by the project leader.

### Outcome measures

All primary and secondary outcome measurements will be carried out at baseline (t0), after each 4-week intervention period (t1, t2, t3, *etcetera*), and before clinical discharge. At baseline, descriptive variables for each subject include age, sex, area and type of infarct, side of stroke, time since stroke onset, body height and body mass.

A summary of the primary and secondary outcome measures can be found in Table 1. The primary outcome will be aerobic capacity, the accepted criterion for exercise capacity [24]. We will estimate standardised cardiopulmonary performance parameters based on international guidelines [25]. Secondary outcome measures will focus on physical performance, stroke impact, and feasibility. We will evaluate the work rate tracking error during FC-RATE by calculating the root mean square error (RMSE) between actual work rate and target work rate values. Gait speed will be assessed using the 10 Meter Walk Test (10MWT) [26,27]. Walking endurance will be measured using the 6 Minute Walk Test (6MWT), the distance that an individual is able to walk in 6 minutes on a hard, flat surface [28,29]. To assess basic standing function in non-ambulatory subjects, we will employ a standardised standing test on a force plate. The subject will be requested to: (1) stand in bipedal stance, (2) shift his/her body weight from the right to the left leg alternately, and (3) shift his/her body weight in a defined rhythm from the right to the left leg alternately. The test-retest reliability of centre of pressure measures in bipedal tasks has been shown previously [30]. We will use the Stroke Impact Scale (SIS) to assess recovery and quality-of-life after stroke [31].

**Table 1:** Overview of primary and secondary outcomes

**Variable**	**Details**	**Unit**
**Primary outcomes (Aerobic capacity)**		
Peak oxygen uptake (VO_2_peak)	Peak oxygen uptake in the last 30 s during IET	mL/min, mL/min/kg
Peak work rate (Ppeak)	Maximal work rate during IET	W
Peak heart rate (HRpeak)	Maximal heart rate during IET	beats/min
Peak ventilation rate (V_E_peak)	Peak ventilation rate in the last 30 s during IET	L/min
Peak respiratory rate (R_f_peak)	Peak respiratory rate in the last 30 s during IET	1/min
Peak respiratory exchange ratio (RERpeak)	Value of the oxygen uptake and carbon dioxide output relationship at peak oxygen uptake	dimensionless
Gas exchange threshold (GET)	Oxygen uptake at the point at which carbon dioxide output begins to increase more rapidly in proportion to oxygen uptake during IET. Estimated using the v-slope method and standard gas exchange criteria	mL/min, mL/min/kg
Oxygen cost of work (ΔVO_2_/ΔP)	Δ oxygen uptake/Δ work rate	mL/min/W
Heart rate response (ΔHR/ΔVO_2_)	Δ heat rate/Δ oxygen uptake	beats/mL/min
Peak oxygen pulse (VO_2_peak/HRpeak)	Peak oxygen uptake/peak heart rate	mL/beat
Time constants of oxygen uptake during on-/off-transition (τ)	Time taken for oxygen uptake to reach 63% of its increment magnitude, when the response is modelled as a mono-exponential function during CLT	s
Steady-state increase in oxygen uptake (ΔVO_2_)	Steady-state increase in oxygen uptake following a step increase/decrease in exercise intensity during CLT	mL/min, mL/min/kg
Steady-state increase in heart rate (ΔHR)	Steady-state increase in heart rate following a step increase/decrease in exercise intensity during CLT	beats/min
Steady-state increase in ventilation rate (ΔV_E_)	Steady-state increase in minute rate following a step increase/decrease in exercise intensity during CLT	L/min
Steady-state increase in respiratory rate (ΔR_f_)	Steady-state increase in respiratory rate following a step increase/decrease in exercise intensity during CLT	1/min
Oxygen cost of work during constant load (VO_2_/P)	Oxygen uptake versus work rate during passive and active constant load phases	mL/min/W
**Secondary outcomes**		
RMS deviation of work rate RMSE_P_	Work rate tracking error during feedback-controlled robotics-assisted treadmill exercise	W
Gait speed	10 Meter Walk Test (10MWT)	m/s
Walking endurance	6 Minute Walk Test (6MWT)	m
Standing function	Standardised protocol to assess standing using a force plate	%, mm
Quality-of-life	Stroke Impact Scale (SIS)	Score
Feasibility	Criteria for success	4 scale rating

CLT, Constant load testing; IET, incremental exercise testing; RMSE, root mean square error.

The feasibility of the study protocol will be based on defined criteria for success such as compliance, attrition, safety, and successful data processing. We define feasibility to be affirmed with compliance to the study protocol of ≥90%, an attrition rate of ≤10%, clinical safety of 100%, and effective data processing of 100%. The overall feasibility of the study will be rated using the following outcomes: (i) protocol deemed feasible (when all criteria for success can be achieved), (ii) protocol deemed feasible with minor modifications (≥75% of the criteria for success can be achieved), (iii) protocol deemed feasible with major modifications (≥50% of the criteria for success can be achieved), and (iv) protocol deemed not feasible (none of the criteria for success can be achieved) [32].

### Instrumentation

**Figure 2:**
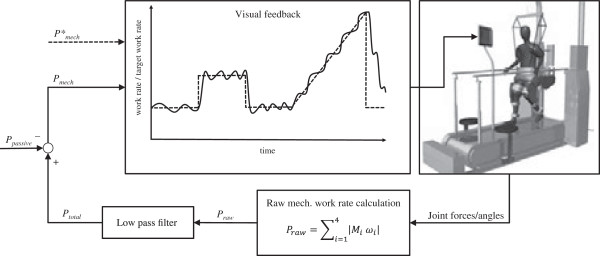
**Feedback-controlled robotics-assisted treadmill exercise.** The solid line represents the mechanical work rate (*P*_*mech*_) produced by the subject. The dashed line represents the target work rate (P*_mech_). The passive mechanical work rate (*P*_*passive*_) is evaluated before every session and subtracted from *P*_*mech*_. Legend: *P*_*raw*_ = raw mechanical work rate, *M*_*i*_ = moments of force, *ω*_*i*_ = angular velocity, *P*_*total*_ = total mechanical work rate.

We will use the Lokomat driven-gait orthosis system (Hocoma AG, Volketswil, Switzerland) integrated with a treadmill (h/p/cosmos sports & medical GmbH, Traunstein, Germany) and a motor-driven body weight support system with real time feedback control for precise body weight unloading (Lokolift, Hocoma AG) to apply RATE. A human-in-the-loop feedback system will control each subject’s active work rate by projecting his mechanical work rate onto a screen at the front of the treadmill together with a target mechanical work rate. Figure 2 illustrates the FC-RATE approach. The subject will be instructed to vary the forces applied on the exoskeleton by volitional muscle activity and to keep the actual work rate as close as possible to the target. This procedure will be used to assess the primary outcome measures (aerobic capacity), and to guide each subject’s individual target work rate during FC-RATE in the experimental group.

Pulmonary gas exchange and ventilatory measurements will be carried out using breath-by-breath spirometry (MetaMax 3B, Cortex Biophysik GmbH, Leipzig, Germany). Prior to each test, a volume calibration will be performed using a standardised volumetric syringe and gas calibration will be carried out using ambient air and a certified precision-analysed gas mixture. Heart rate will be continuously measured and recorded by a heart rate belt (T31, Polar Electro, Kempele, Finland) and a receiver board (HRMI, Sparkfun, Boulder, USA). Blood pressure will be monitored by a sphygmomanometer (HEM- 907, Omron Corporation, Kyoto, Japan). A software module for the overall procedure has been programmed in LabVIEW (Version 2009, National Instruments, Austin TX, USA) [33].

### Testing procedure

The first RATE session will focus on adjusting the device to provide a physiological gait pattern, and on familiarisation with the facemask for respiratory monitoring. An initial test of decreasing body weight support (BWS) continuously by 5% each minute will be carried out to define individual BWS levels. Furthermore, we will implement a short period of incremental exercise to familiarise the subject with the feedback-control structure and to estimate dynamic maximal voluntary contraction during walking (MVC-W) within the exoskeleton to adjust the individual target work rate for the following exercise tests.

**Figure 3:**
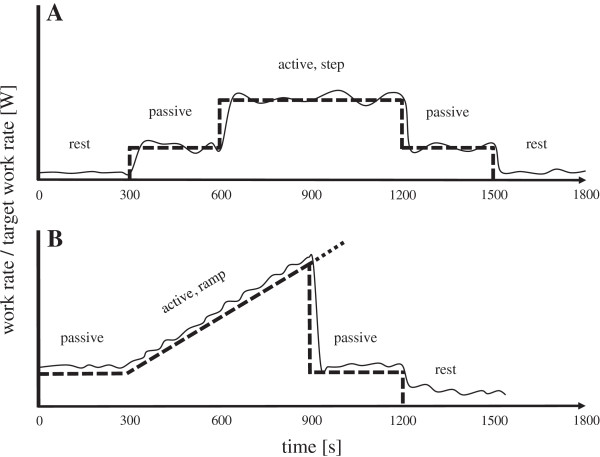
**Exercise testing protocols.** Schematic representation of constant load testing **(A)** and incremental exercise testing **(B)** using feedback-controlled robotics-assisted treadmill exercise. The solid line represents the mechanical work rate (*P*_*mech*_) produced by the subject. The dashed line represents the target work rate (P*_mech_). The slope will be estimated such that the predefined peak work rate is reached after 10 minutes. When individual termination criteria are met the incremental phase is ended and P*_mech_ set back to the passive level.

We will use constant load testing (CLT) [34] to implement a constant work load during RATE. Figure 3A shows a schematic representation of the CLT protocol that consists of: (1) a rest phase where subjects will stand on the treadmill attached to the exoskeleton for 5 minutes with 0% BWS; (2) a passive phase where subjects will walk passively with their individual BWS for 5 minutes; and (3) an active phase where subjects will be instructed to actively contribute to the walking by pushing forward within the exoskeleton during the swing phase of each leg to reach the target work rate, which will be defined as 40% of MVC-W. The target work rate profile will be displayed on a screen in front of the subject. Using real-time visual feedback of their actual work rate the subjects will be asked to maintain their active contribution as close as possible to the target work rate. This phase will be terminated after 10 minutes; (4) passive phase/recovery: subjects will be instructed to walk passively with their individual BWS for 5 minutes; (5) rest: subjects will stand on the treadmill, still attached to the exoskeleton, for 5 minutes with 0% BWS.

Incremental exercise testing (IET) [35] will be used to estimate peak cardiopulmonary performance parameters. Figure 3B shows a schematic representation of the IET protocol that consists of: (1) a passive phase where subjects will be instructed to walk passively with their individual BWS for 5 minutes; (2) and active phase where subjects will be instructed to actively contribute to the walking by pushing forward within the exoskeleton during the swing phase of each leg to reach peak exercise capacity, where the slope will be defined so that MVC-W is reached in approximately 10 minutes; and (3) a passive phase/recovery where subjects will be instructed to walk passively with their individual BWS for 5 minutes.

Both test protocols will follow strict termination criteria for cardiopulmonary exercise testing including: (1) abnormal blood pressure responses, that is, hypertensive (systolic BP >210 mmHg or diastolic BP >115 mmHg) when exercising at high work rate, or hypotensive responses (decrease in BP >10 mmHg) despite an increase in work rate; (2) individual work rate below target work rate for 60 s; (3) peak heart rate within 10 beats per minute of the age-predicted heart rate maximum [36], where the formula will be adjusted down to 70% of heart rate maximum for subjects on β-blocker medications [37]; and (4) pain or discomfort in the chest. We will follow established models for cardiopulmonary exercise testing based on the ACSM guidelines [24].

The 10MWT and the 6MWT will be conducted using standardised testing protocols in a quiet area. Standing function will be assessed within parallel bars. The SIS will be administered to the subjects on a face-to-face basis.

### Intervention

Both groups will receive regular RATE sessions (3/week, 30min/session) during their inpatient rehabilitation period (typically, 4 to 12 weeks). Training intensity will be recorded by continuous heart rate monitoring and evaluating rate of perceived exertion (RPE) on the 6 to 20 scale [38]. The experimental group will receive progressive cardiovascular exercise using FC-RATE. Training will start at 40% of peak work rate (based on initial IET results) for the first session. Intensity will then be adjusted by increasing the target work rate in 5% steps for subsequent sessions based on heart rate and RPE outcomes (target heart rate: 40 to 70% of heart rate reserve, target RPE: 11 to 14). The control group will receive conventional RATE, where therapists will focus on gait quality only. To confirm or refute low cardiovascular training intensity in the control group (target heart rate: ≤30% of heart rate reserve, target RPE: ≤7), therapists will monitor heart rate and RPE during all training sessions. All subjects will receive individually tailored conventional therapy including physiotherapy (4 to 5 sessions/week, 30 to 60min/session), occupational therapy (2 to 3 sessions/week, 30 to 60min/session), and individual speech and language therapy.

### Safety arrangements

There is an inherent risk during cardiopulmonary exercise testing procedures and cardiovascular intervention programmes for subjects with sub-acute stroke of a recurrent cerebral or cardiac event. Furthermore, subjects are attached to the exoskeleton, which complicates emergency procedures. Several risk management arrangements will be implemented to ensure subjects’ safety: (1) clearly defined eligibility criteria to include medically stable subjects only, (2) continuous blood pressure monitoring during exercise testing (noted above), (3) presence of resuscitation-trained assistants, (4) opportunity to call the medical resuscitation team in the clinic, and (5) presence of personnel trained to release the subject within 60 s from the exoskeleton.

### Sample size and power calculation

The required sample size has been calculated based on pooled estimation of standardised effect sizes obtained from a previous meta-analysis on cardiovascular exercise early after stroke [18]. Power analysis was done using G*Power Software (Version 3.1, University of Dusseldorf, Germany). *A priori* analysis indicated that a sample of at least 14 subjects would provide a statistical power of 80%, that is, an 80% (β = 0.2) chance to detect a 20% (α = 0.05) difference between groups. To allow for dropouts, a total of 20 subjects will be recruited.

### Data management and analysis

Data will be recorded on standardised forms and concealed between examiners and therapists. All data recorded continuously during testing will be saved automatically by the software module and will only be accessible by an independent software specialist. Data set examples will be checked for errors randomly or at any time a serious adverse event occurs. Raw breath-by-breath data will be processed using a zero phase shift moving average filter over 15 breaths [39]. Peak cardiopulmonary response variables will be calculated by analysing the final 30 s during incremental exercise, whereby criteria for maximal aerobic capacity will be (1) plateau in oxygen uptake, (2) respiratory exchange ratio (RER) ≥1.15, and (3) peak heart rate within 10 beats per minute of the age-predicted heart rate maximum (adjusted for subjects on β-blocker medications) [24]. Further peak performance variables will be defined as the maximal values at the termination time of the IET. The gas exchange threshold (GET) will be estimated using the v-slope method and standard gas exchange criteria [40]. Step response kinetics will be calculated using a non-linear least-squares algorithm to fit the data as described in the following mono-exponential equation: VO_2(t)_=VO_2_(b)+ΔVO_2_(1-e^-(t-Td)/τ^), t > 0, with VO_2_(b) = oxygen uptake at baseline, ΔVO_2_ = step increase in oxygen uptake, Td = time delay of 20 s corresponding to the cardiodynamic phase of the response, and τ = time constant [34]. Steady-state will be defined as the mean value of the final 120 s during constant load phases. All analyses will be performed using MATLAB (Version R2010a, MathWorks, Natick MA, USA) and MetaSoft (Version 3.9.6 SR1, Cortex Biophysik GmbH, Leipzig, Germany).

### Statistical analysis

Descriptive statistics will be calculated for dependent and independent variables. Demographic and clinical characteristics of the groups will be compared on admission using unpaired two-sample *t*-tests (continuous data) and Chi-square analysis (categorical data). Nonparametric methods will be used when assumptions of normality are violated. To evaluate the clinical efficacy of the method, group differences will be compared after each 4-week intervention (t1, t2, t3, *etcetera*) using unpaired two-sample *t*-tests for continuous data and z-tests for categorical data that conform to the assumption of normality. If data are not normally distributed for continuous variables, Mann-Whitney *U* tests will be applied. A mixed effects analysis of variance with repeated measures to model the intervention effects over time for continuous variables that are normally distributed (that is, aerobic capacity) will be applied. Non-parametric tests (Mann-Whitney *U* and Kruskall Wallis tests) will be used for categorical data (that is, SIS) or where a normal distribution is not present. Variables that are significantly different in the baseline analysis will be used as covariates for further analysis. Multiple regressions will be applied to evaluate relationships among variables. All statistical analysis will be performed using SPSS (SPSS Inc., Chicago, Il, USA). The significance level will be 0.05.

## Discussion

The focus of this pilot trial is to evaluate the efficacy and the feasibility of cardiovascular exercise using FC-RATE with regard to aerobic capacity, motor recovery, and quality-of-life early after stroke. Study results will contribute to the evidence base regarding effects and feasibility of a novel cardiovascular exercise intervention for individuals suffering from severe motor impairment. Furthermore, the results will help the development of further methods for assessment of aerobic capacity and guidance of exercise intensity during early stroke rehabilitation.

It has previously been shown that cardiovascular exercise is effective in improving aerobic capacity and walking distance in individuals with moderate or low impairment after stroke [18]. However, there is a lack of appropriate intervention methods to include highly impaired individuals with low motor status. Chang *et al*. recently explored the effects of RATE on aerobic capacity and motor recovery early after stroke [41]. This conventional approach of reducing BWS and guidance torque, and increasing treadmill speed to guide exercise intensity during RATE showed improved aerobic capacity and increased lower extremity strength in non-ambulatory individuals. However, subjects received 2 weeks of regular exercise only, which is a relatively short aerobic training volume, and the guidance of exercise intensity using this conventional approach was reported to be very difficult.

The present pilot trial will implement a feedback-control structure for guidance of exercise intensity during RATE. This is the first controlled clinical application of this novel concept in an intervention study and might be a first step into intensity-guided cardiovascular rehabilitation research for individuals suffering from severe motor impairment early after stroke. The long-term objective of this research programme is to provide a basis for exploration of the association of early cardiovascular activation/exercise and neural plasticity. There are a few studies that support this objective by reporting promising effects of early cardiovascular exercise on cognition [42], motor function of the lower extremities [43,44], and functional independence [45]. The present trial could provide new insights into cardiovascular rehabilitation early after stroke and may, therefore, guide future research on this topic. This will be relevant for further development of rehabilitation robotics and future stroke rehabilitation strategies.

## Conclusion

Our overall research plan aims to further develop and implement robotics-assisted devices for cardiovascular rehabilitation soon after stroke. The approach presented here has been tested previously in a phase II clinical trial that has shown feasibility of the concept for assessment of aerobic capacity and guidance of cardiovascular exercise in severely impaired individuals. The phase III clinical trial described here aims to determine early indications of the presence and magnitude of efficacy as well as to refine the definition of the experimental protocol. If this trial is able to show reasonable clinical feasibility and to demonstrate having potential for efficacy on the primary outcomes (aerobic capacity), further full-scale trials would be warranted to investigate overall effectiveness of this approach. In case of poor feasibility and low efficacy, the concept might have to undergo further revision and development to optimally support cardiovascular exercise in severely impaired populations.

## Trial status

The trial was designed and commenced in 2012. The expected time of completion of enrolment is June 2014.

## Abbreviations

ACSM: American college of sports medicine; BWS: Body weight support; CLT: Constant load testing; FAC: Functional ambulation category; FC-RATE: Feedback-controlled robotics-assisted treadmill exercise; GET: Gas exchange threshold; IET: Incremental exercise testing; MVC-W: Maximal voluntary contraction during walking; RATE: Robotics-assisted treadmill exercise; RER: Respiratory exchange ratio; RMSE: Root mean square error; RPE: Rate of perceived exertion; SIS: Stroke impact scale; t0: Baseline; t1: retest 1 after 4 weeks; t2: retest 2 after 8 weeks; t3: retest 3 after 12 weeks; VO2peak: Peak rate of oxygen uptake; 6MWT: 6 minute walk test; 10MWT: 10 meter walk test.

## Competing interests

The authors declare that they have no competing interests.

## Authors’ contributions

OS, EDB, RDB and KH were responsible for the design and the methodology of the study. OS and CS accomplished the ethical approval. MS was responsible for the technical development. OS wrote the first draft of this manuscript and was responsible for the revisions. EDB, RDB and KH supervised the process, provided expertise, and critically revised the manuscript. All authors read and approved the final manuscript.
